# Advancing Compatibility and Interfacial Interaction Between PEEK and GNPs Through a Strategic Approach Using Pyrene-Functionalized PDMAEMA-b-PMMA Copolymer

**DOI:** 10.3390/polym17121599

**Published:** 2025-06-08

**Authors:** Chae-Yun Nam, Dohyun Im, Jun-Hyung Lee, Jinwon Kim, Kie-Yong Cho, Ho-Gyu Yoon

**Affiliations:** 1 Department of Materials Science and Engineering, Korea University, 145, Anam-ro, Seongbuk-gu, Seoul 02841, Republic of Korea; namchyoon@korea.ac.kr (C.-Y.N.);; 2 SOLUSYS Co., Ltd., 44-6, Seobong-ro 755beon-gil, Jeongnam-myeon, Hwaseong-si 18522, Republic of Korea; 3 Department of Energy and Chemical Materials Engineering, Pukyong National University, 45 Yongso-ro, Nam-gu, Busan 48513, Republic of Korea

**Keywords:** polyetheretherketone, graphene nanoplatelet, amphiphilic compatibilizer, dispersibility

## Abstract

Polyetheretherketone (PEEK), known for its high heat and chemical resistance and excellent mechanical properties, is extensively utilized, particularly as a metal substitute, in the automotive industry. Although PEEK exhibits outstanding properties, enhancements are essential to improve its practical performance. In this study, we aimed to improve the performance of PEEK by incorporating graphene nanoplatelets (GNPs) and optimizing their dispersion through non-covalent functionalization. We synthesized pyrene-functionalized poly(dimethylaminoethyl methacrylate)-b-poly(methyl methacrylate) (py-PDMAEMA-b-PMMA) as a compatibilizer of PEEK and GNPs and investigated the thermal, mechanical, and tribological properties of the PEEK/GNP composites—GNPs treated with py-PDMAEMA-b-PMMA (F-GNP) and untreated GNPs (pristine GNPs, P-GNP). The F-GNP composites exhibited higher crystallinity and tensile strength than the P-GNP composites, with the best performance observed at a GNP content of 0.1 wt.%. Furthermore, scanning electron microscopy analysis confirmed the enhanced tribological behavior (including a low friction coefficient and reduced abrasive wear) of the F-GNP composites. These enhancements were attributed to the improved interfacial bonding and uniform stress distribution enabled by py-PDMAEMA-b-PMMA. These findings highlight the potential of F-GNP composites to expand the application scope of PEEK to fields requiring superior mechanical performance, such as the automotive and electronics industries.

## 1. Introduction

Polyetheretherketone (PEEK) exhibits excellent tribological performance (specific wear rate ~6 × 10^−6^ mm^3^/Nm) [1], outstanding chemical resistance (resistant to most substances) [2], exceptional thermal stability (glass transition temperature ~143 °C; melting temperature ~343 °C) [3,4], and excellent mechanical properties (e.g., tensile strength 90~110 MPa) [5], making it highly suitable for electrical and electronics industrial applications, as well as for automotive and medical components [6,7]. In particular, PEEK has garnered attention as a metal replacement in automobiles, which require materials with excellent mechanical properties, along with friction and wear characteristics, for long-term use. Although PEEK cannot entirely replace all metallic components, it has proven highly effective as a substitute for specific applications such as thrust washers, gears, and seal rings, particularly in systems where high wear resistance, dimensional stability, and thermal durability are essential [8,9,10,11].

Despite its many advantageous properties, PEEK exhibits relatively low mechanical strength and limited wear resistance under high-load conditions for broader application in structural or friction-intensive environments. As a result, the incorporation of reinforcing fillers has been widely investigated as a means of enhancing its mechanical, thermal, and tribological performance. Incorporating inorganic fillers into PEEK is one of the most effective approaches to improve its properties. Strong and lightweight composites with high performance are produced by combining graphene nanoplatelets (GNPs) and carbon nanotubes (CNTs) with base resins. In particular, GNPs, with their two-dimensional plate-like structures and sp^2^ bonds, impart exceptional thermal stability, mechanical properties, and electrical conductivity to a polymer matrix [12,13,14]. Numerous studies have reported the use of nanocarbons, including GNPs, CNTs, and carbon nanofibers (CNFs), to enhance the mechanical, thermal, and tribological properties of PEEK. Physical mixing, enabling dispersion of CNTs, GNPs, and CNFs, reportedly improves mechanical strength [15,16], and CNTs also demonstrate the ability to enhance thermal resistance [17,18]. However, these studies report challenges associated with agglomeration due to the tendency of nanocarbons to stick together through π––π stacking and van der Waals interactions [19].

In the specific context of GNP-reinforced composites, some critical issues remain unresolved. (1) Inadequate dispersion of GNPs due to their intrinsic tendency to agglomerate, (2) insufficient interfacial adhesion between the GNP and the PEEK matrix, and (3) the lack of thermally stable surface functionalization strategies that preserve the intrinsic sp^2^-conjugated structure of GNPs. Such agglomeration negatively impacts the physical properties of the composites, highlighting the need to functionalize GNPs to enhance their dispersibility and improve the compatibility between the matrix and filler. Functionalized GNPs address these challenges by strengthening interfacial bonding and expanding the surface area for matrix–filler interactions. For example, compatibilizers have been applied to enhance the dispersibility of GNPs, CNTs, and CFs, thus achieving improved mechanical properties [20,21]. Surface treatments using coupling agents have also been employed to further improve the dispersibility of GNPs and CNTs [22]. While these strategies have yielded partial improvements, covalent functionalization approaches such as silane grafting, ethylene-octene elastomer grafted maleic anhydride (PEG-g-MA), and polyaniline (PANi) often disrupt the sp^2^-conjugated structure of GNPs, resulting in the degradation of their intrinsic thermal and mechanical properties [23,24,25]. In addition, the limited thermal stability of these modifiers restricts their applicability in high-temperature engineering environments such as PEEK composites.

To overcome these limitations, a non-covalent functionalization strategy was employed that combines strong π–π interactions with GNP surfaces and matrix compatibility via its amphiphilic architecture. Compared to covalent functionalization with conventional surface modifiers, this approach enables both enhanced dispersion and robust interfacial reinforcement in high-performance thermoplastics such as PEEK, which are capabilities rarely achieved using traditional surface modifiers. Importantly, it is designed to precisely engineer the filler–matrix interface, thereby enhancing the mechanical and tribological performance of the composite without compromising the intrinsic structural integrity of GNPs. Moreover, non-covalent functionalization maintains the aromatic structure and sp^2^ hybridization of GNPs, thereby preserving their inherent stiffness, high modulus, conductivity, and thermal stability.

Building on this concept, we propose employing a non-covalent functionalization strategy to modify GNP by attaching groups that preserve their aromatic structures. Specifically, we synthesized and attached pyrene-functionalized poly(dimethylaminoethyl methacrylate)-block-poly(methyl methacrylate) (py-PDMAEMA-b-PMMA) to improve the surface characteristics of the GNPs and promote better dispersion. Owing to the dual-affinity architecture, py-PDMAEMA-b-PMMA is expected to not only facilitate the stable dispersion of GNPs within the PEEK matrix but also improve interfacial stress transfer through enhanced entanglement with polymer chains. In this design, the pyrene moiety promotes strong π–π interactions with the GNP surface, while the PMMA segment contributes to polymer chain entanglement and compatibility with the PEEK matrix. These interfacial improvements are expected to lead to enhanced crystallization behavior, mechanical strength, and wear resistance compared to conventional P-GNP composites. Coating the GNPs with py-PDMAEMA-b-PMMA improved their compatibility with the matrix, thus enabling their effective dispersion regardless of the matrix polarity. The crystallization behavior was analyzed based on both non-isothermal and isothermal crystallization processes. The activation energy was determined using the Kissinger equation for non-isothermal crystallization, whereas the crystallization half-time was derived from the isothermal crystallization analysis results. The crystallization behavior was further evaluated to investigate its relationship with the mechanical properties, tribological performance, and thermal degradation characteristics of the composites. This comprehensive approach provides insights into the mechanism by which functionalized GNPs (F-GNP) influence the overall performance of PEEK/GNP composites.

## 2. Experimental

### 2.1. Materials

Polyetheretherketone (PEEK), with a molecular weight of approximately 62,000 g/mol, was purchased from Evonik Industries (VESTAKEEP 2000G, Essen, Germany). The PEEK consists of the repeating unit –[–C6H4-O-C6H4-O-C6H4-CO-]–, corresponding to the IUPAC name poly(oxy-1,4-phenylenecarbonyl-1,4-phenyleneoxy-1,4-phenylene). The resin is an unfilled, high-performance, engineering-grade polymer widely applied in demanding industrial applications. Graphene nanoplatelets (GNP, R-25 grade) were purchased from XG Science, East Lansing, MI, USA. The GNPs exhibit a flake-like morphology with an average lateral size of approximately 2.76 μm × 2.07 μm and a thickness of 12.7 nm, as observed by transmission electron microscopy (TEM), corresponding to ~37 layers based on an interlayer spacing of 0.34 nm. The specific surface area ranges from 30 to 60 m^2^/g. To synthesize an amphiphilic diblock copolymer as a compatibilizer, 2-(dodecylthiocarbonothioylthio)propionic acid (97%) and 2,2′-azobis(2-methylpropionitrile) (AIBN, 99%) were employed as the chain transfer agent and initiator, respectively. Further, 2-(dimethylamino)ethyl methacrylate (DMAEMA, 98%) and methyl methacrylate (MMA, 99%) were used as monomers, and 1-(bromomethyl)pyrene (95%) was used to synthesize py-PDMAEMA-b-PMMA. Tetrahydrofuran (THF, 99%), N,N-dimethylformamide (DMF, >99%), and chloroform (CF, 99.5%) were used as solvents (Samchun Chemical Co., Ltd., Pyeongtaek, Republic of Korea). All other chemicals used for the synthesis of py-PDMAEMA-b-PMMA were purchased from Sigma Aldrich Co., Ltd., St. Louis, MO, USA.

### 2.2. Synthesis of py-PDMAEMA-b-PMMA

The detailed synthesis is described in our previous report [26]. In this study, py-DMAEMA-b-PMMA, an amphiphilic compatibilizer, was synthesized using the reversible addition fragmentation chain transfer technique. The chain transfer agent (CTA, 2-(dodecylthiocarbonothioylthio)propionic acid) and AIBN were placed in a round-bottom flask and stirred under vacuum to remove moisture from the surface. Then, toluene was added, and the mixture was stirred at room temperature until the CTA and AIBN were fully dissolved. After purging the solution with argon gas, the purified DMAEMA monomer was added to the CTA and AIBN mixture, and the solution was placed in an oil bath for approximately 16 h. The second monomer, MMA, was added while continuing to purge with argon gas, and the final polymerization proceeded in an oil bath for 24 h. The resulting diblock copolymer, PDMAEMA-b-PMMA, was diluted with tetrahydrofuran (THF) and deposited in a small amount of n-hexane. The sediment was filtered through a 0.2-µm Teflon filter. To remove any unreacted monomer, the filtered PDMAEMA-b-PMMA was redissolved in THF, and the filtration process was repeated three times. After the final filtration, the obtained PDMAEMA-b-PMMA was dried in a vacuum oven. To attach pyrene to PDMAEMA-b-PMMA, the latter was dissolved in N,N’-dimethylformamide (DMF); next, 1-(bromomethyl)pyrene was added to the solution, which was then allowed to react for 24 h at room temperature. The product was purified using hexane, and the final py-PDMAEMA-b-PMMA product was dried at room temperature in a vacuum oven.

### 2.3. Surface Treatment of GNPs with py-PDMAEMA-b-PMMA

First, 10 mg of pristine GNPs were added to 90 mL of chloroform and sonicated for 10 min. Next, 20 mg of py-PDMAEMA-b-PMMA mixed in 10 mL of chloroform was added to the GNP-dispersed solution. The mixture was then sonicated for 30 min and stirred overnight. The solution was filtered through a 0.2-μm teflon filter to remove unreacted py-PDMAEMA-b-PMMA. This washing process was repeated twice to ensure thorough purification.

### 2.4. Preparation of PEEK/GNP Composites

Compounding was performed using a micro twin extruder. In the first step, the GNPs were dry-mixed with PEEK, extruded using a twin extruder, and then subjected to pelletization. In the second step, the pellets obtained from the first process were melt-mixed again through the extruder to ensure better distribution of the GNPs. The pellets were again pelletized, and the final pellets were used for further processing.

### 2.5. Measurements

To assess the thermal properties and crystallinity of the composites, differential scanning calorimetry (DSC) (DSC 25, TA Instruments, New Castle, DE, USA) analysis was performed. A comparative analysis of crystallinity was conducted using X-ray diffraction (XRD; SmartLab, Rigaku, Auburn Hills, MI, USA). The mechanical properties were evaluated using a universal testing machine (UTM, 4467, INSTRON Co., Norwood, MA, USA) in accordance with ASTM D638. Tensile tests were performed on at least seven dog-bone-shaped specimens per sample group, and the results were averaged with the standard deviation calculated. To characterize the synthesized py-PDMAEMA-b-PMMA, thermogravimetric analysis (TGA; Q600, TA Instruments, New Castle, DE, USA) was performed under a nitrogen atmosphere at a heating rate of 10 °C/min. ^1^H nuclear magnetic resonance (NMR; VNMRS500, Varian Inc., Palo Alto, CA, USA) spectroscopy analysis was performed with CDCl_3_ as the solvent. Raman spectroscopy (LabRam ARAMIS IR2, Horiba Jobin Yvon SAS, Paris, France) analysis was performed using a 785 nm diode laser to achieve a spatial resolution of 1 μm at 100× magnification, with a 0.26 mm gap between the sample and lens. Gel permeation chromatography (GPC; EcoSEC HLC-8320, Tosoh, Tokyo, Japan) was conducted using a system equipped with a refractive index detector. Tetrahydrofuran (THF) was used as the eluent at a flow rate of 1.0 mL/min, and the system was calibrated using polystyrene standards. For validation of the GNP characterization data, Raman measurements were performed twice, whereas NMR, GPC, and TGA analyses were each repeated three times to ensure experimental consistency and reproducibility. The morphology of the samples was examined using scanning electron microscopy (SEM; LYRA3 instrument, TESCAN BRNO SRO, Brno-Kohoutovice, Czech Republic) and transmission electron microscopy (TEM; Talos F200X instrument, Thermo-Fisher Scientific, Waltham, MA, USA). For TEM analysis, approximately 1 mg of the F-GNP was dispersed in 5 mL of ethanol and subjected to bath sonication for 10 min to promote uniform dispersion. A small aliquot of the dispersion was drop-cast onto a copper TEM grid and air-dried at room temperature prior to observation. Tribological measurements were performed using a pin-on-disk tribometer (THT, Anton Paar, Graz, Austria) in accordance with ASTM G99.

## 3. Results and Discussion

### 3.1. Functionalization of py-PDMAEMA-b-PMMA on GNPs

**Figure 1:**
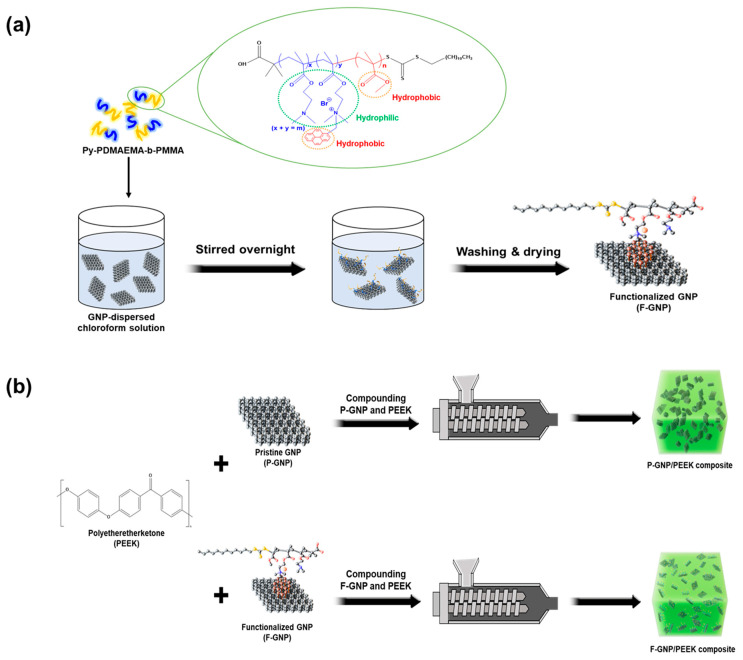
(**a**) Schematic illustration of the functionalization process of GNPs with py-PDMAEMA-b-PMMA and (**b**) fabrication of P-GNP/PEEK and F-GNP/PEEK composite via melt compounding through a twin-screw extruder.

GNP agglomeration disrupted their uniform dispersion within the polymer matrix, thus affecting the performance of the composites. To address this issue, GNPs functionalized with py-PDMAEMA-b-PMMA, offering high compatibility regardless of polarity, were synthesized. The pyrene moiety in py-PDMAEMA-b-PMMA was strongly attached to the surface of the GNPs through non-covalent π–π interactions, preserving the structural integrity of the GNPs. Additionally, py-PDMAEMA-b-PMMA showed π–π interactions with the GNPs and entangled with the PEEK matrix, thus enhancing the dispersion and compatibility of the GNPs. This functionalization strategy promoted the uniform distribution of the GNPs throughout the composite, thereby improving their mechanical and thermal properties. A schematic representation of the overall process is shown in Figure 1.

### 3.2. Characterization of py-PDMAEMA-b-PMMA

**Figure 2:**
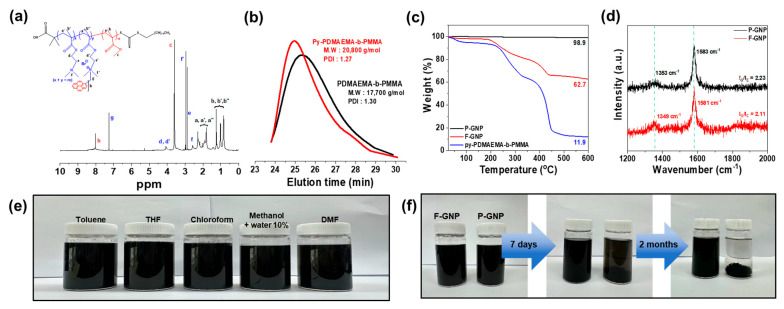
(**a**) ^1^H−NMR spectra of py−PDMAEMA−b−PMMA and (**b**) GPC data of PDMAEMA-b-PMMA and py−PDMAEMA−b−PMMA. (**c**) TGA and (**d**) Raman spectra of P−GNP and F−GNP. (**e**) Photographs of F−GNP solutions in toluene, THF, chloroform, methanol, and DMF. (**f**) Long−term dispersion stability of F−GNP and P−GNP in chloroform.

Successful functionalization of the pyrene moieties in PDMAEMA-b-PMMA was confirmed by ^1^H NMR analysis (Figure 2a). Peaks corresponding to the methylene (e and d) and dimethyl protons of the PDMAEMA functional group (f) were observed. Their shifts to e′ and f′, respectively, indicated the interaction of these protons with the tertiary amine following pyrene addition. The unique peaks (h and g), observed only for py-PDMAEMA-b-PMMA, were attributed to pyrene. Additionally, a peak was observed at ~8.0 ppm corresponding to the pyrene moieties at the terminus of PDMAEMA, further validating the functionalization process. TGA provided additional insights into the functionalization and thermal stability of the GNPs. The GPC results revealed that py-PDMAEMA-b-PMMA had a molecular weight of 20,800 g/mol and a polydispersity index of 1.27 (Figure 2b). The estimated number of pyrene units per chain was 10.4 with an m:n of approximately 3:7. Pristine GNP (P-GNP) exhibited minimal decomposition, retaining 98.9% of its residue at 600 °C (Figure 2c). In contrast, py-PDMAEMA-b-PMMA underwent decomposition, leaving only 11.9% of the residue. F-GNP retained 62.7% of its residue, indicating that approximately 36.2% of py-PDMAEMA-b-PMMA was successfully attached to the GNP surface. This functionalization was further confirmed using Raman spectroscopy [26,27]. P-GNP exhibited peaks at 1581 cm^−1^ (G band) and 1349 cm^−1^ (D band) (Figure 2d). In contrast, F-GNP showed a slight redshift, with peaks at 1583 cm^−1^ (G band) and 1353 cm^−1^ (D band). A distinct redshift in both the D and G bands was observed for F-GNP compared to P-GNP, which is indicative of altered local electronic environments at the graphene surface [28]. This shift is attributed to π–π stacking interactions between the pyrene moieties of the block copolymer and the sp^2^-hybridized carbon framework of GNP, along with potential interlayer coupling effects induced by physical adsorption [29,30]. The redshift of the G band, in particular, signifies the establishment of strong π–π electronic interactions, confirming the effective anchoring of the compatibilizer to the GNP surface [31]. Moreover, the largely unchanged D band intensity suggests that the sp^2^-conjugated carbon structure of GNP is preserved, thereby verifying that the non-covalent functionalization proceeds without introducing significant structural defects [23,32]. These results collectively validate the efficacy and structural fidelity of the non-covalent approach employed in this work [33]. This result was consistent with the findings of Yeom et al. and Zahid et al., who demonstrated that because of their broad planar aromatic structures, compatibilizers strongly bind to the hydrophobic surface of GNPs through π–π interactions without disrupting the electronic conjugation of the GNP structure [34,35].

The dispersion stability of F-GNP in solvents with varying polarity indices is shown in Figure 2e. The selected solvents were toluene (2.4), THF (4.0), chloroform (4.1), DMF (6.4), and methanol with 10% water (9.0); this range covered both nonpolar (toluene) and polar (methanol + water) solvents. The dispersion stability of F-GNP was higher than that of P-GNP across all the solvents. These results aligned with the findings of Cho et al., who enhanced the dispersibility of multiwalled CNTs (MWCNTs) by attaching an amphiphilic compatibilizer [36,37]. To assess long-term dispersion stability, we compared the behaviors of P-GNP and F-GNP in chloroform over two months (Figure 2f). F-GNP maintained stable dispersion throughout the observation period, whereas P-GNP began settling within seven days and showed complete sedimentation by the end of two months. These results demonstrate that the F-GNP composite shows improved immediate and long-term stabilities in various solvent environments.

### 3.3. Analysis of py-PDMAEMA-b-PMMA Functionalization Through TEM

**Figure 3:**
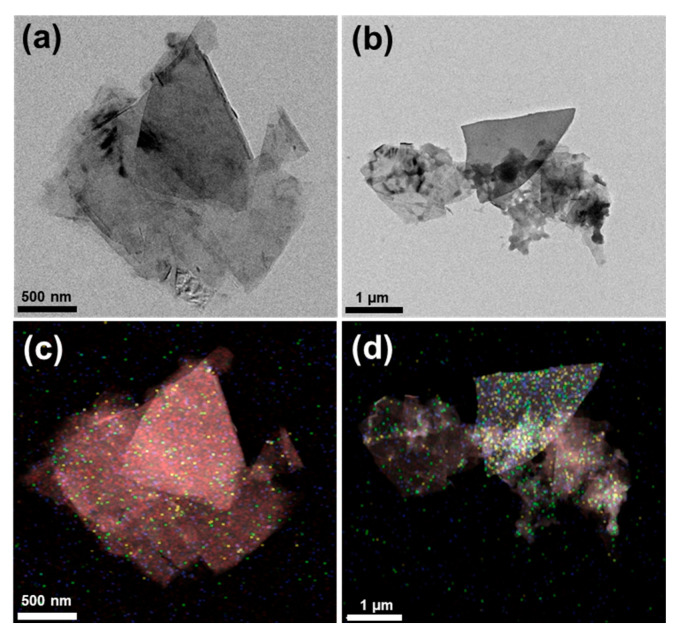
FETEM images of (**a**) P-GNP and (**b**) F-GNP, and EDS mapping images obtained from TEM analysis of (**c**) P-GNP and (**d**) F-GNP. The elemental distribution is represented as follows: carbon (red), nitrogen (green), oxygen (blue), and sulfur (yellow) (individual EDS maps for each element are provided in Figures S1 and S2).

In Figure 3b, the small dots visible in the F-GNP sample are attributed to the nanosized PDMAEMA domains formed by the py-PDMAEMA-b-PMMA functionalization. This observation aligns with the results of previous studies, such as that of Yang et al., who identified similar rounded nanosized PDMAEMA domains in their samples containing PDMAEMA attached to graphene oxide through TEM analysis [38]. Figure 3c,d present energy-dispersive X-ray spectroscopy (EDS) images of P-GNP and F-GNP, respectively. The elemental mapping corresponds to the distribution of carbon (C, red), nitrogen (N, green), oxygen (O, blue), and sulfur (S, yellow). These elements originate from the chemical structure of the py-PDMAEMA-b-PMMA compatibilizer used to functionalize the GNP surface. Specifically, carbon and oxygen arise from the methacrylate backbone and ester functionalities in both PMMA and PDMAEMA blocks. Nitrogen is associated with the tertiary amine groups in PDMAEMA. Sulfur is derived from the RAFT agent residue used during synthesis. The P-GNP image shows minimal amounts of nitrogen, oxygen, and sulfur. In contrast, the F-GNP image reveals a wide distribution of these elements, confirming the successful attachment of py-PDMAEMA-b-PMMA to the GNP surface. The increased concentrations of nitrogen, oxygen, and sulfur in F-GNP further validated the effectiveness of the functionalization process, demonstrating that py-PDMAEMA-b-PMMA was uniformly applied to the GNPs.

### 3.4. Thermal Behavior and Crystallinity of the P-GNP and F-GNP Composites

**Figure 4:**
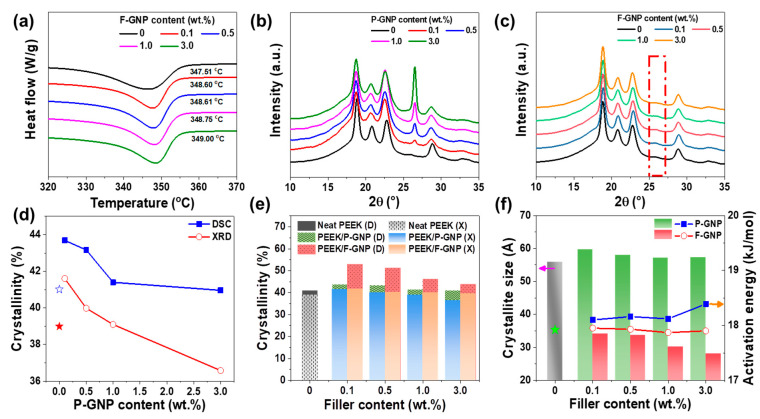
(**a**) T_m_ for F-GNP composites at a heating rate of 10 °C/min; XRD patterns of (**b**) P-GNP composites and (**c**) F-GNP composites as a function of GNP content. (**d**) Comparison of crystallinity of P-GNP composites, obtained through DSC and XRD; (**e**) comparison of crystallinity of P-GNP and F-GNP composites measured by DSC and XRD, respectively (D: DSC and X: XRD). (**f**) Crystallite size and activation energy according to GNP content. (In (**f**), the pink arrow indicates the bar graph corresponding to crystalline size on the left *y*-axis, while the orange arrow denotes the symbol-line graph representing activation energy on the right *y*-axis. The green star marks the activation energy at 0 wt.%.)

The melting temperature (T_m_) increased with increasing GNP content within the PEEK composites (Figure 4a) owing to the GNP-induced restricted PEEK chain mobility, which limited the movement of the polymer chains and required high energy, resulting in a high T_m_. These findings are consistent with those of Alvarado et al., who reported that T_c_ increased by 5 °C upon the addition of 1 wt.% GNP in PEEK [39]. Although the F-GNP exhibited improved dispersion within the PEEK matrix, the corresponding T_g_ enhancement was moderate (Figure S3 and Table S1) [30]. This modest increase observed in F-GNP composites may result from a higher fraction of rigid amorphous phase near the improved dispersed GNP. This locally restricts chain mobility without reducing the overall amorphous content, which may explain the sharper T_g_ in Figure S3. The enhanced interfacial interactions likely promote rigid amorphous phase formation with minimal impact on the mobile amorphous phase. This can be attributed to the non-covalent nature of the interactions and the relatively low filler loading, which, while sufficient to enhance interfacial compatibility, may not impose a strong enough constraint on PEEK segmental motion to induce a large T_g_ shift [40]. In addition, the increased crystallinity observed upon F-GNP incorporation may further reduce the amorphous fraction, thereby contributing to the modest overall change in T_g_.

Figure 4b,c shows the XRD patterns of the P-GNP composites and F-GNP composites, respectively, revealing an increase in the intensity of the peak at 26.47° with increasing GNP content. This peak corresponds to the characteristic reflection of GNPs, and the increased peak intensity confirms the increase in GNP content in the P-GNP composites [41]. In contrast, the XRD pattern of the F-GNP composite does not display a peak, suggesting that the enhanced dispersion of the GNPs achieved through functionalization suppresses the GNP agglomeration. This observation aligns with the findings of Cho et al., who demonstrated that non-covalent functionalization of GNPs in a polyketone matrix could lead to exfoliation, leading to an interlayer spacing that eliminated the GNP peak [42]. Additionally, peaks observed were detected at 2θ = 18.8°, 20.7°, 22.9°, and 28.9°, corresponding to the (110), (111), (200), and (211) lattice planes of the orthorhombic unit cell of PEEK, respectively.

The crystallinities of the P-GNP and F-GNP composites, measured by DSC and XRD, differed owing to the distinct principles of these methods. DSC is a thermal analysis technique that measures heat flow during phase transition, capturing both crystalline and amorphous regions. Because heating enhances macromolecular mobility, this technique induces partial crystallization before melting, and the corresponding observation results incorporate contributions from both phases, often resulting in higher crystallinity values. In contrast, XRD analyzes materials without altering their structures. XRD selectively detects diffraction from crystalline regions. However, quantification of crystallinity using this technique involves a comparison between crystalline peaks and the amorphous background. As a result, the resulting crystallinity values are generally lower than those determined using DSC [43].

The crystallinity determined by DSC is calculated using the following formula:

(1) $${\;X}_{C}(\%)=\frac{{∆H}_{m}}{\emptyset \times {∆H}_{m}^{\infty }}\times 100$$

where ${∆H}_{m}$ is the melting enthalpy of the sample obtained using DSC, ${∆H}_{m}^{\infty }$ is the enthalpy of the PEEK crystal (130 J/g) [44], and *Φ* represents the weight fraction. Both the P-GNP and F-GNP composites exhibited the highest crystallinity at a GNP content of 0.1 wt.%; however, the crystallinity decreased as the GNP content increased (Figure 4e and Table 1). At a heating rate of 10 °C/min, the crystallinity of the P-GNP composite increased to 43.69%, whereas that of the F-GNP composite, with 0.1 wt.% GNP, was 53.04%; this result was attributed to the GNPs that served as effective nucleation agents in both the composites, thus promoting crystal growth. However, as the GNP content increased further, the mobility of the PEEK chains became restricted, which hindered further crystal growth [45]. A similar phenomenon was observed by Al-Saleh et al., who reported that when GNPs were added to polypropylene (PP), the nucleation effect was prominent up to a certain level, beyond which the GNPs restricted the polymer chain’s movement and hindered further crystal growth [24].

Crystallinity is determined from the XRD patterns using the following relationship:

(2) $$X_{c}=\frac{A_{c}}{A_{c}+A_{a}}$$

where $A_{c}$ and $A_{a}$ are the areas of the crystalline and amorphous peaks, respectively [43]. The high specific surface area of the GNPs influenced the chain mobility within the PEEK matrix. At high GNP contents, particularly beyond 1.0 wt.%, the reduced chain mobility restricted the formation of well-ordered lamellae, thereby hindering effective crystallization (Figure 4d). Consequently, crystallinity reduced at higher GNP loads (measured by XRD), specifically in the case of the P-GNP composites, in which the limited dispersion and weak interactions of the GNPs with the matrix further reduced the crystallinity. In addition, the XRD analysis revealed differences between the crystallinities of the P-GNP and F-GNP composites. The F-GNP composites exhibited the highest crystallinity (41.72%) at 0.1 wt.% GNP, and the value then gradually decreased upon further increasing the GNP content. The observed enhanced crystallinity of the F-GNP composites could be attributed to the presence of additional crystal-formation sites due to the more uniform distribution of the GNPs within the PEEK matrix. Across all the investigated GNP contents, the crystallinity of F-GNP remained consistently higher than that of P-GNP. This increase in crystallinity was primarily due to the improved dispersion of the GNP facilitated by py-PDMAEMA-b-PMMA, which ensured a uniform distribution of nucleation sites throughout the matrix. The crystallinity of the P-GNP composites decreased when the GNP content exceeded 0.5 wt.%, whereas the F-GNP composites maintained their high crystallinity, which even exceeded that of the 0 wt.% GNP composites.

Next, we analyzed the isothermal crystallization kinetics of the PEEK/GNP composites to determine the crystallization half-time (τ12) from the point where 50% crystallization was achieved. Typically, a high τ12 value signifies a low crystallization rate, indicating a prolonged crystallization process. We observed the τ12 value of the F-GNP composites was higher than that of the P-GNP composites (Figure S4 and Table S2). This result indicated that the improved dispersibility of F-GNP increased the number of effective nucleation sites, thus facilitating crystallization initiation and decelerating the overall crystallization process. Additionally, F-GNP enhanced the PEEK chain’s mobility and lowered the activation energy. Although the activation energy for crystallization was reduced in the F-GNP composites, isothermal DSC analysis revealed a longer crystallization half-time (τ1/2), indicating a slower but more coordinated molecular rearrangement. This behavior, observed under controlled thermal conditions, led to the formation of crystals with higher structural order and fewer defects [46,47]. It should be noted that these conditions are not representative of industrial processing, where crystallization occurs over much shorter timescales. Nonetheless, the isothermal experiments provide fundamental insight into how improved nucleation from well-dispersed F-GNP can promote more orderly crystal growth, which supports the observed enhancement in composite properties. Calculations performed using the Scherrer equation indicated that the crystallite size of the F-GNP composite was smaller than that of the P-GNP composite (Figure 4f, Figure S5 and Table S3). The enhanced dispersibility of F-GNP increased the area of the interface with PEEK, thus increasing the number of nucleation sites and reducing the crystallite sizes owing to growth interference. The extended crystallization time resulted in a high degree of crystal perfection [48]. Despite their small crystallite sizes, the F-GNP composites attained high crystallinity owing to the presence of abundant nucleation sites. In contrast, the P-GNP composites formed large crystallites with gaps between them, leading to the formation of an amorphous region [49]. Consequently, the P-GNP composites exhibited a less ordered crystal structure with weak intermolecular bonding. Because the deformability of P-GNP was higher than that of F-GNP, it was more susceptible to failure under external stress and directly reduced the mechanical strength while increasing the wear rate of the corresponding composites [50]. Furthermore, during thermal aging, the F-GNP composites maintained a high degree of crystallinity and superior mechanical strength owing to their robust crystal structures.

### 3.5. Morphology Analysis of the Composites According to GNP Surface Treatment

**Figure 5:**
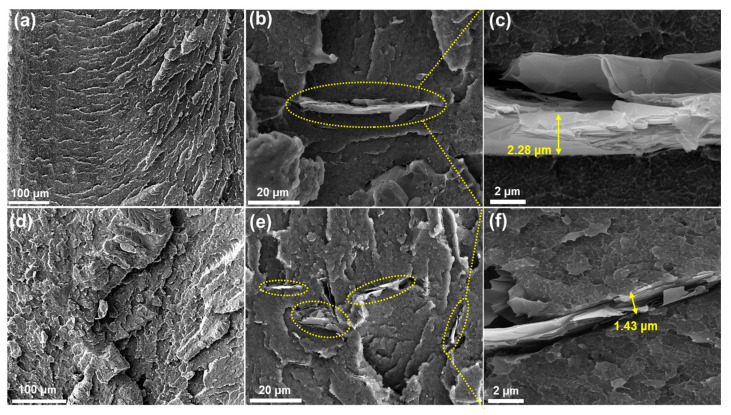
SEM images of the fractured surfaces of 1.0 wt.% filled P-GNP (**a**–**c**) and F-GNP (**d**–**f**) composites (listed by image magnification).

Figure 5a,d depict the fracture surfaces obtained at 1 wt.% GNP. During fracture, the material absorbs energy, generating numerous microcracks that result in the formation of severely cracked fracture surfaces [51,52,53]. As shown in Figure 5, the fracture surface of the F-GNP composite (Figure 5d) exhibits a rougher and more irregular morphology compared to that of the P-GNP composite (Figure 5a). This increased roughness is attributed to stronger interfacial bonding between the F-GNP and the PEEK matrix, promoting tortuous crack propagation and energy dissipation during fracture [54]. In contrast, the smoother and more planar fracture surface of the P-GNP composite suggests brittle failure resulting from weak filler-matrix adhesion. A comparison of Figure 5b,e demonstrates that py-PDMAEMA-b-PMMA improves dispersion through non-covalent interactions, leading to the formation of agglomerates with sizes smaller than those of the P-GNP composites. Figure 5c,f further confirm this observation, showing that the P-GNP composites almost retain their original thickness, whereas the F-GNP composites show notably smaller clusters owing to the improved dispersion.

### 3.6. Mechanical Properties of the GNP Composites

**Figure 6:**
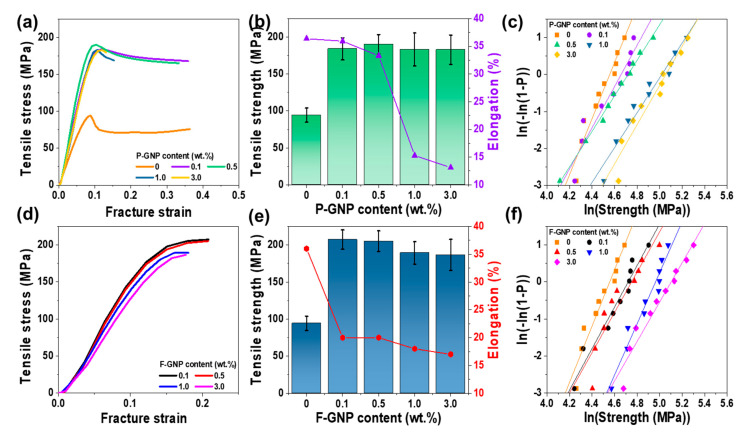
(**a**,**d**) Tensile stress–strain curves of P−GNP and F−GNP composites. (**b**,**e**) Tensile strength and elongation of P−GNP and F−GNP composites. (**c**,**f**) Weibull distribution of P−GNP and F−GNP composites.

Figure 6 shows the tensile properties of P-GNP (a–c) and F-GNP (d–f) (multiple stress-strain curves for each GNP content in both P-GNP and F-GNP composites, Figures S6 and S7). The P-GNP composites exhibited typical necking behavior independent of the GNP content due to the weak interaction between PEEK and the GNP, showing the heterogeneous surfaces, which reflect the lack of continuity between the GNP and PEEK matrix. Consequently, the tensile properties were predominantly governed by the PEEK matrix. This result highlighted the limited ability of P-GNP composites to maintain structural integrity under stress caused by insufficient interfacial bonding, which allowed deformation prior to failure [54]. The P-GNP composites exhibited an ultimate tensile strength of neat PEEK of 94.33 MPa with a strain of 0.36 (Figure 6b). The tensile strength initially increased with increasing P-GNP content, maximizing at 0.5 wt.%.

The fracture strain of the P-GNP composites was higher than that of the F-GNP at 0.1 and 0.5 wt.% GNP content. In contrast, the F-GNP composites showed markedly reduced strains at high GNP contents primarily because the enhanced interaction and entanglement between the PEEK matrix and F-GNP increased the ductility and brittleness of these composites. Furthermore, the tensile strength of the P-GNP composites was lower than that of the F-GNP composites across all GNP contents. This reduced tensile strength was attributed to the formation of agglomerates, which acted as stress concentrators and initiated cracks that hindered the ability of the composites to effectively distribute and dissipate stress. Durmas et al. reported similar trends, showing that 0.5 and 1.0 wt.% GNP addition to poly(lactic acid) increased the tensile strength of the resulting material by 10% and by 5%, respectively, relative to that of the neat resin [55]. Our results indicated that due to the functionalization, the mechanical properties of the F-GNP composites remained superior to those of the P-GNP composites at all investigated GNP contents. Specifically, the tensile strength of the F-GNP composites increased to 207.27 MPa at 0.1 wt.% GNP, surpassing that of the P-GNP composites. This improvement in tensile strength was accompanied by strain reduction, with the F-GNP composites exhibiting a fracture strain of 0.20 at 0.1 wt.% GNP. This reduction in fracture strain could be ascribed to the enhanced entanglement between PEEK and the GNPs and highly efficient stress transfer throughout the composites due to the improved dispersion of GNPs within the PEEK matrix. B.D. Sahm et al. and H. Ejaz et al. reported that the presence of agglomerations not homogeneously dispersed in the PEEK matrix results in structural defects [56,57] and that this improvement of tensile strength was accompanied by a reduction in fracture strain, indicating a trade-off between stiffness and ductility [51,58]. The superior performance of the F-GNP composites, both in terms of tensile strength and fracture strain reduction, is attributed to their improved interfacial bonding, high degree of entanglement, and highly efficient stress distribution. The incorporation of F-GNP increased the brittleness of the composite, which could also be associated with the observed increase in mechanical strength (Figure 5). High energy is required for crack propagation through this rough interface because the strong interfacial bonding and entanglement hinder crack initiation and growth, thereby improving the ability of the composite to absorb energy under mechanical stress [59]. The strong interfacial interaction prevents localized deformation, leading to suppression of the necking behavior typically observed in ductile materials. This trend was consistent with the findings of Domun et al., who reported that 1.0 wt.% F-GNP addition to epoxy resulted in high tensile strength [60]. Additionally, Lee et al. demonstrated that GNP exfoliation, caused by compatibilizer treatment in PP resin, improved interfacial adhesion and effective stress transfer, further confirming the mechanical benefits of F-GNP composites [61]. Moreover, the stress–strain curves of the F-GNP composites further emphasized their mechanical superiority; evidently, the tensile strength of these composites increased, whereas the fracture strain values decreased with increasing GNP content. This improvement could be attributed to the py-PDMAEMA-b-PMMA treatment, which facilitated better entanglement between PEEK and the GNPs, resulting in more efficient stress transfer throughout the composite. Because of these enhanced interactions, the F-GNP composites exhibited consistently higher tensile strength and lower strain values (Table 2). The improved Young’s modulus observed in F-GNP composites can be primarily attributed to enhanced interfacial stress transfer facilitated by the py-PDMAEMA-b-PMMA (Figure S8) [62]. In addition, for F-GNP, such interfacial reinforcement plays a more decisive role in modulus enhancement than the intrinsic stiffness of the filler itself [63]. The mechanical enhancement in F-GNP composites can be attributed to multiple reinforcing mechanisms, including improved load transfer efficiency through enhanced interfacial adhesion, crack bridging, and deflection effects by well-dispersed GNPs and an increase in matrix crystallinity [51,57,64,65]. These synergistic contributions are consistent with the observed increase in tensile strength, particularly at optimal F-GNP contents. The increased crystallinity, along with the formation of well-aligned chains and crystals, further reinforced the mechanical properties by enhancing structural integrity and optimizing the load distribution within the PEEK matrix. While the reduction in ductility may suggest increased brittleness, such behavior can be advantageous in high-performance structural applications where mechanical reliability, dimensional stability, and resistance to deformation under load are critical. This is particularly relevant for components in the automotive, aerospace, and various industries, where composites with higher mechanical properties and consistent fracture behavior are preferred over those exhibiting excessive plastic deformation. The tensile strength of the PEEK composites was analyzed using the Weibull distribution method; the shape parameter (*β*) was derived from the slope of the Weibull plot (Figure 6c,f). Typically, a high *β* value indicates a narrow distribution of strength data, reflecting the high reliability of composites. We observed that the *β* value of the F-GNP composites was higher than that of the P-GNP composites. This result suggested that the F-GNP showed both improved dispersion and compatibility within the PEEK matrix. Notably, this enhanced uniformity in the composites ultimately contributed to increasing the reliability of the F-GNP composites.

### 3.7. Tribological Properties of the Composites According to Functionalization

**Figure 7:**
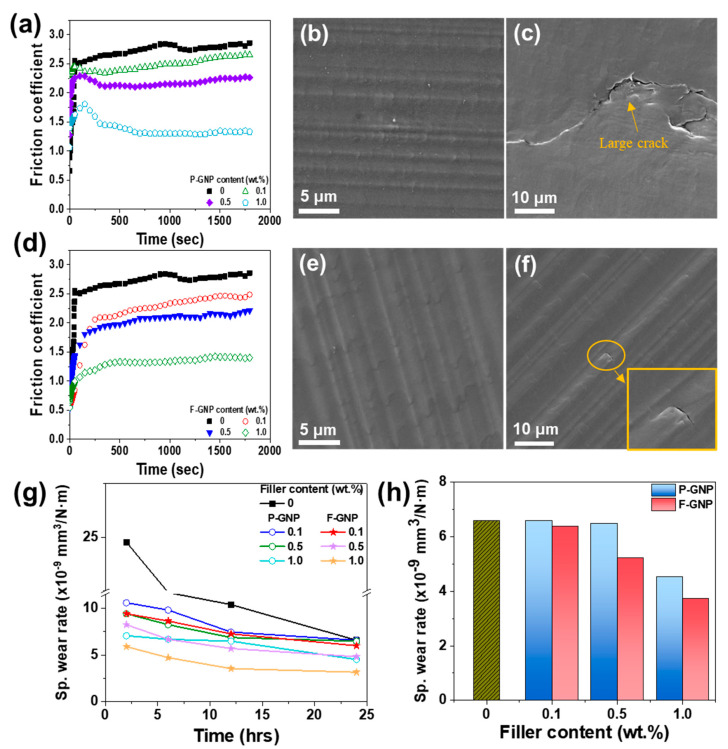
Friction coefficient of (**a**) P−GNP and (**d**) F−GNP composites. SEM images of the worn surface in the wear track of (**b**) P−GNP and (**e**) F−GNP and cracks in (**c**) P−GNP and (**f**) F−GNP composites at 1.0 wt.% GNP content. (**g**) Time-dependent specific wear rate of P−GNP and F−GNP composites, and (**h**) specific wear rate of the composites measured after 24 h of sliding.

The tribological performance of the composites was primarily evaluated based on their friction coefficients and wear resistance. The friction coefficient decreased as the P-GNP and F-GNP contents increased (Figure 7a,d) owing to the lubricating effect of the GNPs, which formed a protective layer on the sliding surface, thereby minimizing direct contact between the PEEK matrix and the pin and ultimately reducing friction [41]. Notably, the friction coefficient of F-GNP was lower than that of P-GNP, primarily owing to the enhanced dispersion of F-GNP, which resulted in a more uniform stress distribution and minimized localized stress concentration within the PEEK matrix. The improved compatibility between F-GNP and the PEEK matrix strengthened the interactions between them, further amplifying the lubricating effect of the GNPs. However, the difference between the friction coefficients of the P-GNP and F-GNP composites was relatively minor. The surfaces of the friction tracks did not exhibit significant differences; this observation further explained the negligible variation in the friction coefficients between the two composites (Figure 7b,e). Figure 7g,h show the specific wear rates for both the P-GNP and F-GNP composites as functions of the filler content, measured after 24 h of sliding under a load of 15 N. The specific wear rate is calculated using the following equation [66]:

(3) $$W_{s}=\frac{∆m}{F\cdot L}$$

where $W_{s}$ is the specific wear rate (mm^3^/N·m), F is the applied load, L is the length of the sliding distance, and ∆m is the mass reduction in the composites. Neat PEEK exhibited a specific wear rate of approximately 6.6 × 10^−9^ mm^3^/N·m. For P-GNP and F-GNP, the specific wear rate generally decreased, reflecting the reinforcing effect of the GNPs on the composites’ wear resistance [67]. The specific wear rates of the P-GNP and F-GNP composites showed notable differences, which could be attributed to the interaction between the PEEK matrix and GNPs. In the P-GNP composites, the weak interaction between P-GNP and the PEEK matrix resulted in poor continuity and weak interfacial adhesion within the composites [68]. Consequently, the inherent properties of the PEEK matrix dominated, even within the composites; this result is evident from Figure 7c, which reveals that large cracks form because of insufficient interfacial bonding. Conversely, the F-GNP composites benefited from the functionalization, which enhanced the interaction and entanglement between the GNPs and PEEK matrix, leading to strong interfacial bonding and improved continuity in the composites. Consequently, GNP detachment and wear were reduced during the wear test (demonstrated by the small cracks visible in Figure 7f) [69]. Ultimately, F-GNP incorporation led to strong interfacial adhesion and, thus, improved the overall interfacial properties and reduced wear. These results demonstrate that although both P-GNP and F-GNP exhibit a friction-reducing effect, the superior performance of F-GNP in terms of wear resistance is correlated to its improved dispersion, strong interfacial bonding, and the formation of a more stable tribolayer.

### 3.8. Thermal Aging Analysis of the P-GNP and F-GNP Composites

**Figure 8:**
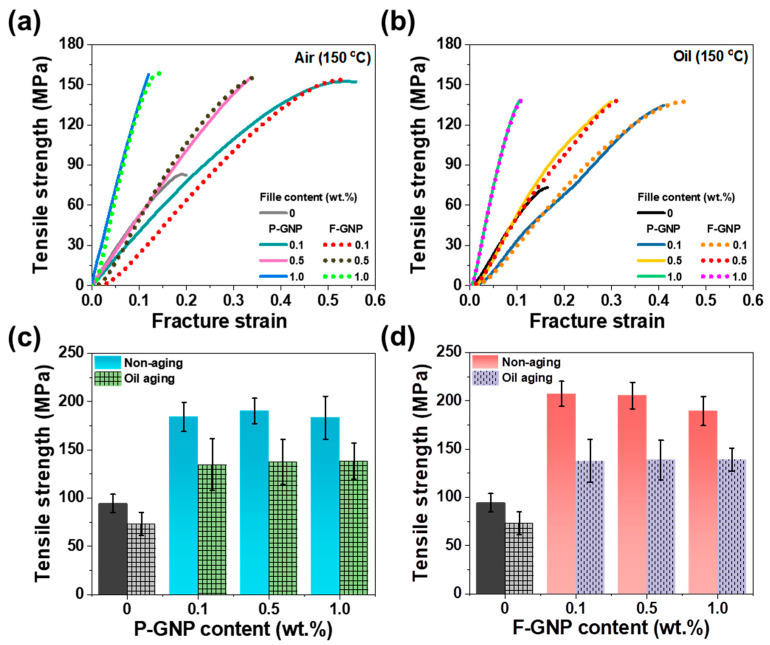
Mechanical properties of P-GNP and F-GNP composites after aging at 150 °C. Stress–strain curves for (**a**) air-aged composites and (**b**) oil-aged composites. Tensile strength of (**c**) P-GNP composites and (**d**) F-GNP composites as a function of GNP content under nonaging and oil-aging conditions. (In Figure 8c,d, the solid dark gray bar represents the tensile strength of the non-aged composite at 0 wt.%, while the light gray bar with pattern indicates the tensile strength of the oil-aged composite at 0 wt.%.)

Automotive components, such as seal rings, thrust washers, and backup rings, utilize PEEK composites because of their excellent durability at elevated temperatures and resistance to oil exposure; these two features are crucial for preventing oil leaks during transmission and ensuring reliable performance during continuous operation. To assess their applicability in automotive environments, aging tests were conducted at 150 °C to replicate harsh environmental conditions. In addition, to evaluate the impact of long-term exposure (as these components are designed to withstand and minimize oil leakage during operation), both air aging and oil-immersion aging tests were performed. Figure 8a,b show the curves of the P-GNP and F-GNP composites after 10 days of exposure to the two different aging conditions, respectively. Under oil aging, the P-GNP composites showed performance deterioration due to oil-infiltration-induced GNP agglomeration and oxidation, which hindered load transfer between P-GNP and PEEK [70,71,72,73]. In contrast, F-GNP demonstrated superior dispersibility, showing a uniform distribution, thus suppressing the formation of weak points or stress concentrators. This enhanced distribution improved the mechanical stability of the composites, even under oil immersion conditions.

A comparison between the results shown in Figure 6 and Figure 8 indicates that both air and oil aging result in tensile strength reduction. However, the strength reduction caused by oil aging is greater than that resulting from air aging owing to oil infiltration, which weakens the interactions between the GNPs and the PEEK matrix. Consequently, the interfacial interactions and entanglements are weakened, and thus, less force is required to disrupt them. Both air and oil aging reduced crystallinity over time, and thus, the crystallinity observed after air and oil aging was lower than that of the unaged samples (Figure S9 and Table S4). This reduced crystallinity weakened the intermolecular bonding, which in turn allowed deformation to occur at low strength due to the presence of less-oriented polymer chains. After both air and oil aging, F-GNP composites exhibited slightly higher tensile strength compared to P-GNP composites. However, the overall difference remained relatively small. This limited improvement is attributed to the low GNP content, where the reinforcing effect from improved interfacial adhesion in F-GNP is not fully maximized [74]. Aging induces chain scission and oxidation in the PEEK matrix, which affects mechanical performance [75,76]. Although F-GNP offers better initial interfacial interaction, prolonged aging can partially degrade the interface, thereby reducing the performance gap between F-GNP and P-GNP composites.

Table 3 outlines the results of the Weibull distribution analysis of the tensile strength data for both aging conditions. Notably, the *β* values of the F-GNP composites exceed those of the P-GNP composites, implying enhanced uniformity in the data distribution (Figure S10). These findings demonstrated that functionalization promoted high filler dispersibility within the matrix, resulting in improved mechanical properties and high resistance to harsh conditions. The entanglement between F-GNP and the polymer matrix impeded oil infiltration. Consequently, the F-GNP composites retained their mechanical integrity more effectively than the P-GNP composites under oil immersion, demonstrating superior oil resistance. These results highlight the potential of F-GNP as a superior reinforcement filler for applications requiring mechanical stability under harsh conditions.

## 4. Conclusions

This study demonstrates that the non-covalent functionalization of GNPs using an amphiphilic block copolymer enhances the overall performance of PEEK-based composites. The pyrene moieties promote π–π interactions with the GNPs, while the PMMA segments enable chain entanglement and improve compatibility with the PEEK matrix. These synergistic interfacial effects enhance filler dispersion and stress transfer without compromising the structural integrity of the nanofillers. More importantly, this approach presents a generally applicable interfacial design strategy in which both strong surface interaction and matrix compatibility play critical roles in improving the functional performance of polymer nanocomposites. This framework offers valuable insight for the development of high-performance composites incorporating nanocarbon fillers and engineering thermoplastics.

The incorporation of GNPs influenced the crystallization behavior of PEEK, with enhanced crystallinity observed at low filler contents due to the nucleating effect. However, excessive loading induced agglomeration and hindered further crystal growth. Notably, F-GNP composites exhibited higher crystallinity than P-GNP composites across all loadings, attributed to improved dispersion from non-covalent functionalization. The increase in crystallinity observed in the F-GNP composites contributes to higher tensile strength by enhancing the load transfer efficiency within the PEEK matrix [4,65]. Furthermore, higher crystallinity improves surface hardness and wear resistance, leading to reduced friction coefficient and wear [11]. These improvements in crystallinity were closely mirrored in the mechanical and tribological properties. While P-GNP composites showed strength reduction at high loadings due to poor dispersion, F-GNP composites maintained superior tensile strength and exhibited lower wear rates and friction coefficients. SEM observations confirmed smoother wear surfaces and fewer defects in F-GNP specimens. In addition, F-GNP composites demonstrated excellent long-term stability under both air and oil aging, further validating the effectiveness of the surface functionalization strategy.

In conclusion, this work demonstrates that non-covalent functionalization with py-PDMAEMA-b-PMMA effectively addresses the dispersion and compatibility limitations associated with pristine GNPs, leading to improvements in the crystallinity, mechanical strength, and tribological properties of PEEK composites. These results highlight the potential of this approach for developing advanced polymer nanocomposites for use in high-performance engineering applications such as automotive components, electronic housings, and tribological parts operating under demanding thermal and mechanical environments.

## Figures and Tables

**Table 1:** Summary of the crystallinity of the P-GNP and F-GNP composites, with varying GNP contents, measured by DSC and XRD.

GNP Content (wt.%)	DSC		XRD	
	P-GNP	F-GNP	P-GNP	F-GNP
0	41.01		39.99	
0.1	43.69	53.04	41.61	43.79
0.5	43.17	51.34	39.98	43.22
1.0	41.40	46.18	39.10	40.55
3.0	40.96	43.83	36.58	39.65

**Table 2:** Summary of mechanical properties of the GNP composites at different GNP contents, measured by a UTM. The shape parameter (*β*) derived from Weibull distribution analysis as a function of GNP content.

GNP Content (wt.%)	Ultimate Tensile Strength (MPa)		Fracture Strain		Shape Parameter (*β*)	
	P-GNP	F-GNP	P-GNP	F-GNP	P-GNP	F-GNP
0	94.33 (±9.66)		0.36		7.60	
0.1	184.01 (±14.99)	207.27 (±13.06)	0.35	0.20	5.58	5.61
0.5	190.26 (±13.07)	205.20 (±13.76)	0.33	0.20	4.81	5.44
1.0	183.13 (±22.31)	189.31 (±15.02)	0.15	0.18	4.71	6.90
3.0	182.89 (±19.82)	186.58 (±20.97)	0.13	0.17	5.31	5.32

**Table 3:** Shape parameter (*β*) of P-GNP and F-GNP composites, derived from Weibull distribution analysis, as a function of GNP content after air aging and oil-immersion aging at 150 °C.

GNP Content (wt.%)	Shape Parameter (*β*)			
	Air Aging		Oil-Immersion Aging	
	P-GNP	F-GNP	P-GNP	F-GNP
0	7.19		6.24	
0.1	7.44	10.26	3.41	4.49
0.5	6.03	14.22	4.08	5.29
1.0	5.62	25.50	5.18	9.90

## Data Availability

Data are contained within the article.

## Supplementary Materials

The following supporting information can be downloaded at https://www.mdpi.com/article/10.3390/polym17121599/s1, Figure S1: Energy-dispersive X-ray spectroscopy (EDS) elemental mapping images of the P-GNP. Elemental distributions are shown for (a) carbon (C, red), (b) nitrogen (N, green), (c) oxygen (O, blue), and (d) sulfur (S, yellow); Figure S2: Energy-dispersive X-ray spectroscopy (EDS) elemental mapping images of the F-GNP. Elemental distributions are shown for (a) carbon (C, red), (b) nitrogen (N, green), (c) oxygen (O, blue), and (d) sulfur (S, yellow); Figure S3: Glass transition temperature (T_g_) of (a) P-GNP composites and (b) F-GNP composites as a function of GNP content; Table S1: Glass transition temperature (T_g_) of P-GNP composites and F-GNP composites as a function of GNP content; Figure S4: Crystallization rate constant (k) of (a) P-GNP and (b) F-GNP composites derived from isothermal crystallization behavior with various temperature and GNP content; Table S2: Kinetic parameters from the analysis of isothermal crystallization of PEEK/GNP composites; Figure S5: XRD patterns of (a) P-GNP and (b) F-GNP composites as a function of GNP content; Table S3: FWHM of the peaks at 2θ values 22.9° and 28.9°; Figure S6: Stress-strain curves of P-GNP composites with varying P-GNP contents: (a) 0 wt.%, (b) 0.1 wt.%, (c) 0.5 wt.%, (d) 1.0 wt.%, and (e) 3.0 wt.%; Figure S7: Stress-strain curves of P-GNP composites with varying P-GNP contents: (a) 0 wt.%, (b) 0.1 wt.%, (c) 0.5 wt.%, (d) 1.0 wt.%, and (e) 3.0 wt.%; Figure S8: Young’s modulus of P-GNP composites and F-GNP composites as a function of GNP content; Figure S9: Crystallinity of P-GNP and F-GNP composites under different aging conditions after 10 days of aging. (The values in brackets, (A) and (O), represent aged in air and oil, respectively.); Table S4: Melting temperature and crystallization temperature of aged composites under different aging conditions; Figure S10: Weibull distribution for air aged (a) P-GNP, (b) F-GNP composites, and oil aged (c) P-GNP and (d) F-GNP composites after 10 days of aging. Reference [77] is cited in the Supplementary Materials.

## Abbreviations

CTA, chain transfer agent; DSC, differential scanning calorimetry; EDS, energy-dispersive X-ray spectroscopy; PEEK, polyetheretherketone; SEM, scanning electron microscopy; TEM, transmission electron microscopy; TGA, thermogravimetric analysis; UTM, universal testing machine; XRD, X-ray diffraction; GPC, gel permeation chromatography.
