# Supplementary material for: Advancing Compatibility and Interfacial Interaction Between PEEK and GNPs Through a Strategic Approach Using Pyrene-Functionalized PDMAEMA-b-PMMA Copolymer
Source: Polymers (Basel). 2025 Jun 8;17(12):1599. doi: 10.3390/polym17121599 (PMC12196970; doi:10.3390/polym17121599)
Supplement: Supplementary file 1 [file polymers-17-01599-s001.zip › polymers-3665987-supplementary.pdf]

Advancing compatibility and interfacial interaction between PEEK and GNP through a strategic approach using pyrene functionalized PDMAEMA-b-PMMA copolymer

Chae Yun Nam[a], Dohyun Im[a], Jun Hyung Lee[a], Jinwon Kim[b], Kie Yong Cho[c] and Ho Gyu Yoon[a,*]

[a] Department of Materials Science and Engineering, Korea University, 145, Anam-ro, Seongbuk-gu, Seoul 136-713, Republic of Korea
[b] SOLUSYS Co.,Ltd., 44-6, Seobong-ro 755beon-gil, Jeongnam-myeon, Hwaseong-si, Gyeonggi-do, Republic of Korea
[c] Department of Energy and Chemical Materials Engineering, Pukyong National University, 45 Yongso-Ro, Nam-Gu, Busan 48513, Republic of Korea.

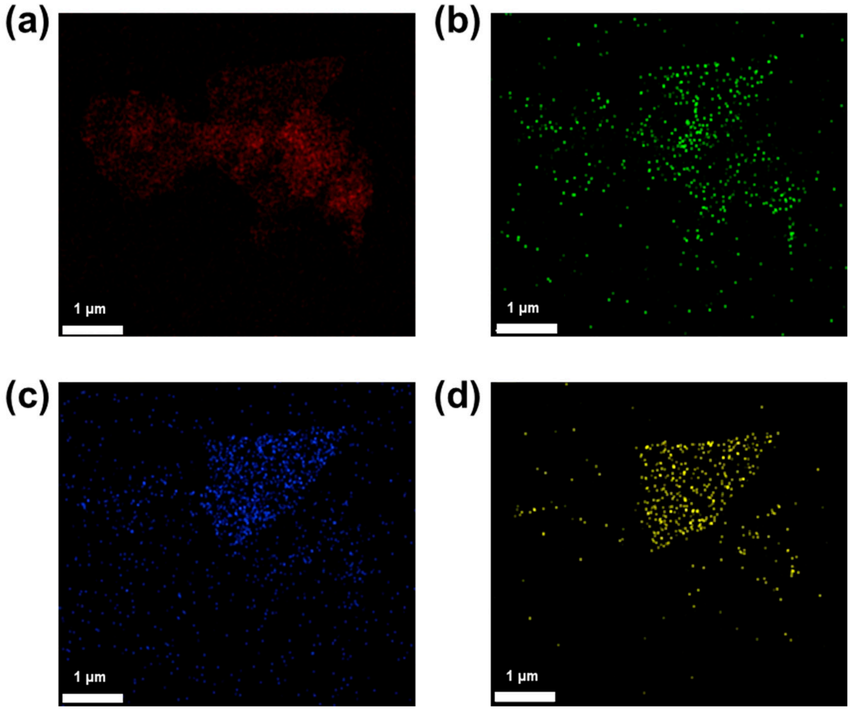

Fig. S1. Energy-dispersive X-ray spectroscopy (EDS) elemental mapping images of the P-GNP. Elemental distributions are shown for (a) carbon (C, red), (b) nitrogen (N, green), (c) oxygen (O, blue), and (d) sulfur (S, yellow).

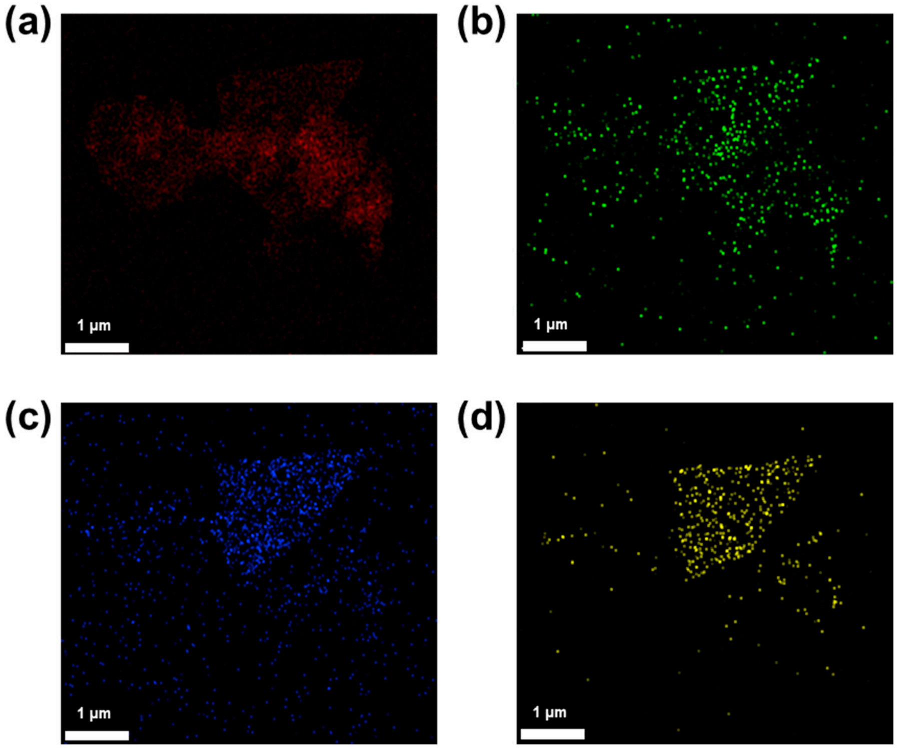

Fig. S2. Energy-dispersive X-ray spectroscopy (EDS) elemental mapping images of the F-GNP. Elemental distributions are shown for (a) carbon (C, red), (b) nitrogen (N, green), (c) oxygen (O, blue), and (d) sulfur (S, yellow).

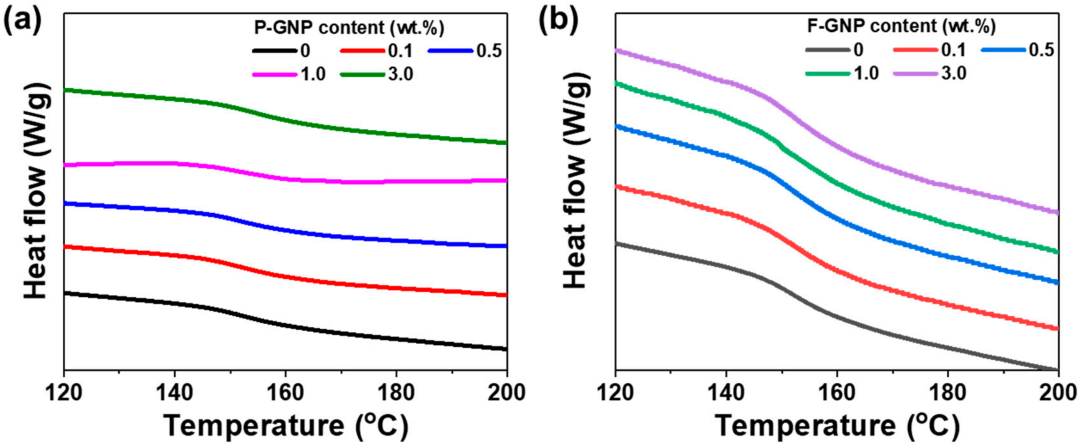

Fig. S3. Glass transition temperature (T_g) of (a) P-GNP composites and (b) F-GNP composites as a function of GNP content.

Table S1. Glass transition temperature (T_g) of P-GNP composites and F-GNP composites as a function of GNP content.

Filler content (wt.%)	T_g (°C) P-GNP	T_g (°C) F-GNP
0	155.13	
0.1	155.38	155.54
0.5	155.58	155.84
1.0	155.69	156.13
3.0	156.33	157.25

As GNP was incorporated into PEEK matrix, the extent of segmental motion restriction is moderate [2]. This leads to slight of modest T_g enhancement, rather than a significant shift [3]. Moreover, the enhanced crystallinity reduces the amorphous fraction and limits the available free volume, which can counteract the anticipated increase T_g arising from improved filler-matrix interactions. This is attributed to the fact that crystalline domains promote chain regularity and impose inherent structural constraints, thereby diminishing the extent to which additives can further influence the segmental dynamics of the polymer matrix.

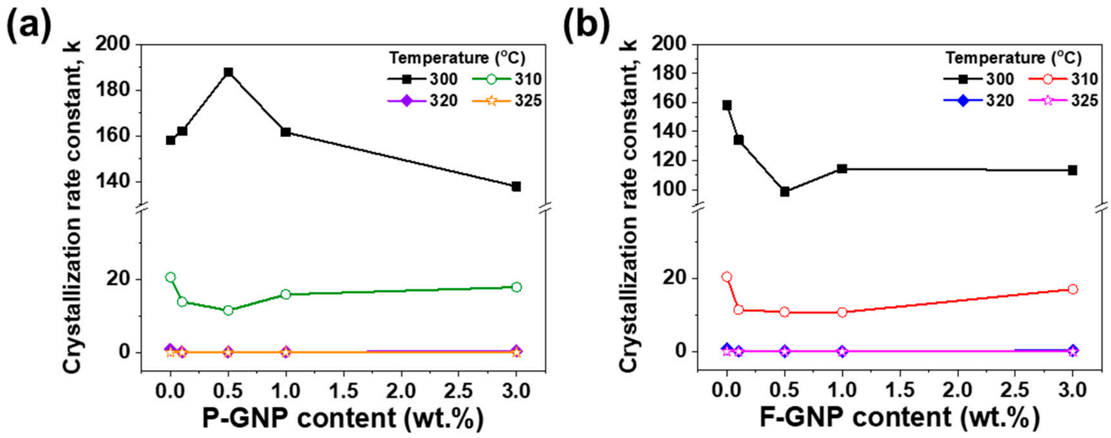

Fig. S4. Crystallization rate constant (k) of (a) P-GNP and (b) F-GNP composites derived from isothermal crystallization behavior with various temperature and GNP content.

Table S2. Kinetic parameters from the analysis of isothermal crystallization of PEEK/GNP composites.

GNP type	Isothermal temperature (°C)	GNP content (wt.%)	n	k	$\tau_{1/2}$
		0	2.45	158.16	0.11
		0.1	2.61	162.10	0.12
	300	0.5	2.48	188.03	0.11
		1.0	2.46	161.62	0.11
		3.0	2.34	138.04	0.10
		0	2.54	20.50	0.26
		0.1	3.01	13.83	0.37
	310	0.5	3.48	11.45	0.45
		1.0	3.11	15.81	0.37
P-GNP		3.0	2.21	17.80	0.23
		0	2.59	0.79	0.95
		0.1	3.19	0.11	1.78
	320	0.5	3.17	0.11	1.76
		1.0	3.28	0.10	1.80
		3.0	2.37	0.39	1.27
		0	3.46	0.01	3.40
		0.1	3.56	0.002	5.17
	325	0.5	3.76	0.003	5.42
		1.0	3.81	00.001	5.57
		3.0	2.58	0.01	5.17
		0	2.45	158.16	0.11
		0.1	2.67	134.32	0.14
F-GNP	300	0.5	2.58	96.65	0.14
		1.0	2.74	114.44	0.16
		3.0	2.40	113.41	0.12
	310	0	2.54	20.50	0.26

	0.1	3.19	11.46	0.42
	0.5	3.15	10.83	0.42
	1.0	3.14	10.75	0.42
	3.0	2.64	17.05	0.30
	0	2.59	0.79	0.95
	0.1	3.33	0.11	1.74
320	0.5	3.21	0.10	1.84
	1.0	3.53	0.05	2.11
	3.0	2.35	0.31	1.41
	0	3.46	0.01	3.40
	0.1	3.89	0.001	5.37
325	0.5	3.22	0.001	5.70
	1.0	4.17	0.0003	6.41
	3.0	2.50	0.01	5.45

P-GNP composites exhibit higher crystallization rate constant and lower crystallization half-time compared to F-GNP composites. This suggests that F-GNP composites undergo a slower crystallization process, resulting in a more well-ordered and structurally perfect crystalline phase compared to P-GNP composites.

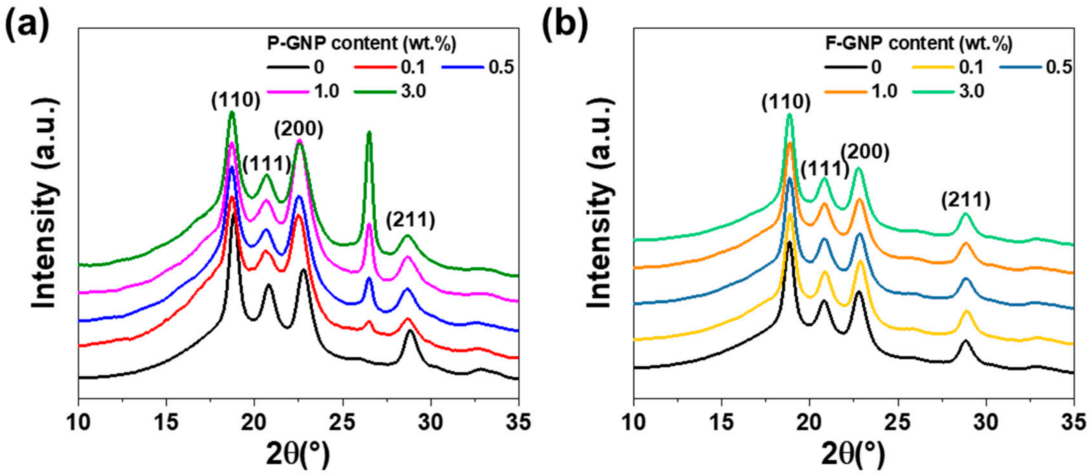

Fig. S5. XRD patterns of (a) P-GNP and (b) F-GNP composites as a function of GNP content.

Table S3. FWHM of the peaks at 2θ values 22.9° and 28.9°.

Filler content (wt.%)	FWHM (22.9°) (radian)		FWHM (28.9°) (radian)	
	P-GNP	F-GNP	P-GNP	F-GNP
0	5.36		5.03	
0.1	5.87	4.37	5.84	3.38
0.5	5.80	3.89	5.25	2.88
1.0	5.75	3.67	5.18	2.35
3.0	5.37	3.32	4.62	1.87

F-GNP composites exhibit reflections along the (200) and (211) planes, which are 22.9° and 28.9°, respectively, with lower intensity compared to P-GNP composites. The (200) plane undergoes growth through widening, while the (211) plane grows through lenghtening and stacking. This results indicated that F-GNP composites have a smaller crystallite growth in both thickness and width [1].

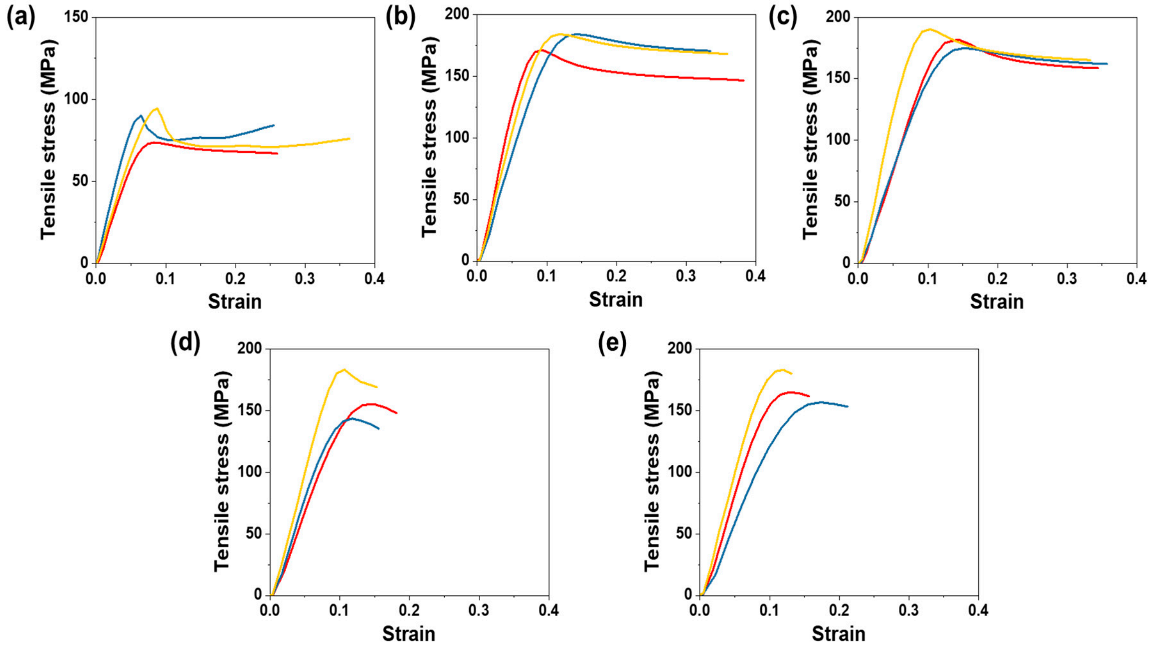

Fig. S6. Stress-strain curves of P-GNP composites with varying P-GNP contents: (a) 0 wt.%, (b) 0.1 wt.%, (c) 0.5 wt.%, (d) 1.0 wt.%, and (e) 3.0 wt.%.

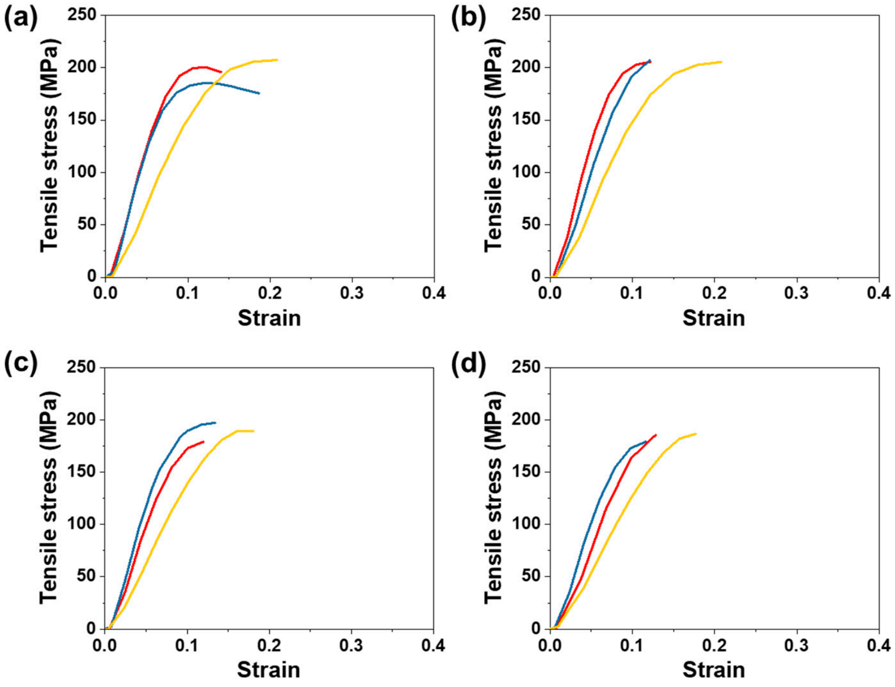

Fig. S7. Stress-strain curves of P-GNP composites with varying P-GNP contents: (a) 0 wt.%, (b) 0.1 wt.%, (c) 0.5 wt.%, (d) 1.0 wt.%, and (e) 3.0 wt.%.

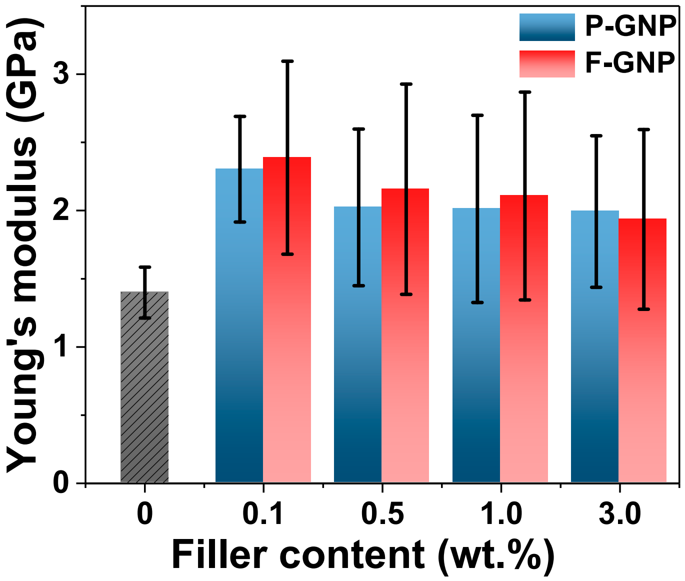

Fig. S8. Young's modulus of P-GNP composites and F-GNP composites as a function of GNP content.

The py-PDMAEMA-b-PMMA functionalization creates a non-covalent yet robust interfacial interaction that promotes enhanced load transfer from the PEEK matrix to the GNP. This occurs via π-π interactions between the pyrene group and GNP surface and polymer chain entanglement between the copolymer blocks and the PEEK matrix, resulting in more effective stress dissipation under tensile loading [4,5].

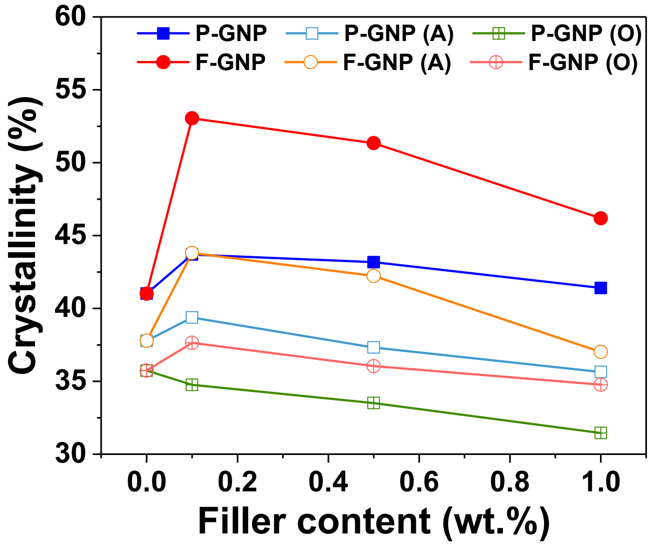

Fig. S9. Crystallinity of P-GNP and F-GNP composites under different aging conditions after 10 days of aging. (The values in brackets, (A) and (O), represent aged in air and oil, respectively.)

Table S4. Melting temperature and crystallization temperature of aged composites under different aging conditions.

GNP type	Filler content (wt.%)	Air aging			Oil aging		
		T_m (°C)	T_c (°C)	X_c (%)	T_m (°C)	T_c (°C)	X_c (%)
P-GNP	0	344.24	304.35	37.78	343.80	303.75	35.73
	0.1	345.29	304.50	39.37	344.07	304.61	34.75
	0.5	345.36	304.65	37.32	344.78	304.68	33.50
	1.0	345.10	304.85	35.64	344.99	304.81	31.45
F-GNP	0.1	345.26	304.55	46.80	344.89	304.01	37.63
	0.5	345.49	304.62	42.22	345.19	304.35	36.04
	1.0	345.22	304.66	37.01	345.22	304.50	34.77

Crystallinity of both air and oil aged samples was decreased over time. Oil aged samples exhibited a lower degree of crystallinity, melting temperature and crystallization temperature,

as the penetration of oil into the composites weakened intermolecular bonding. The higher crystallinity of F-GNP observed in both air aged and oil aged samples indicates that F-GNP exhibits greater reliability even under harsh conditions.

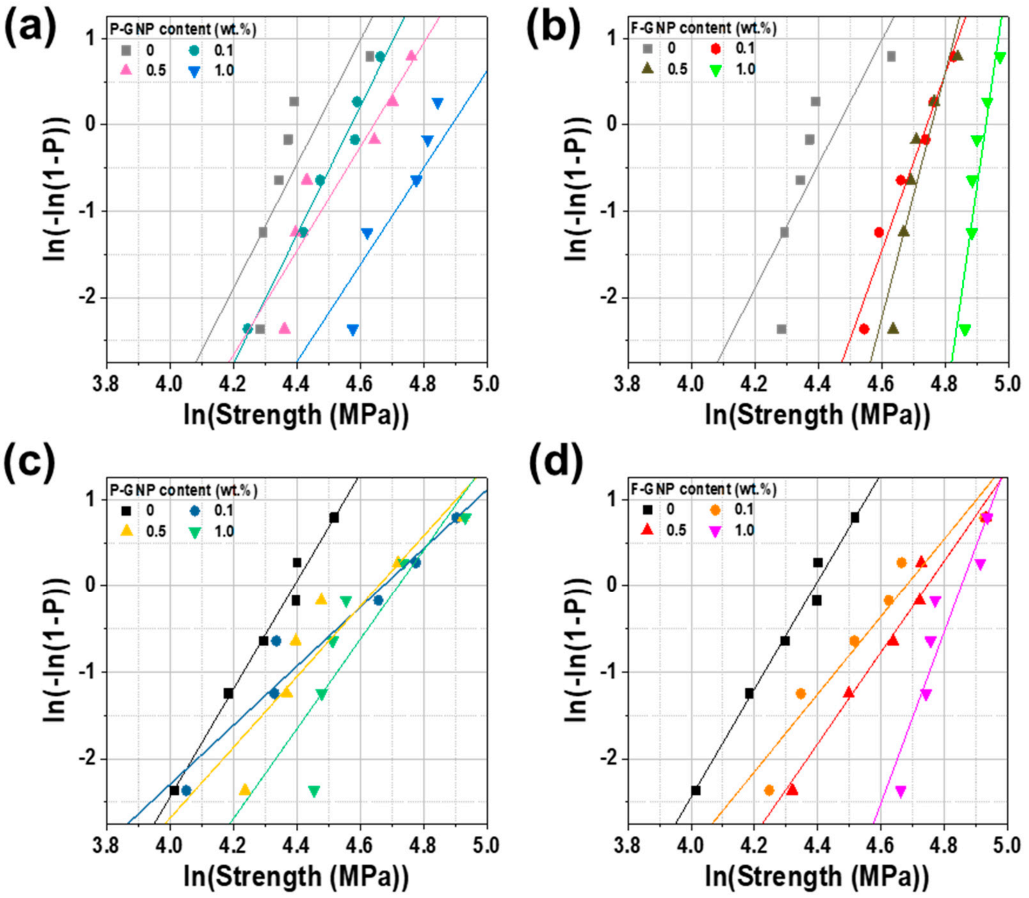

Fig. S10. Weibull distribution for air aged (a) P-GNP, (b) F-GNP composites, and oil aged (c) P-GNP and (d) F-GNP composites after 10 days of aging.

Fig. S4. supports the Table 3. in manuscript. Shape parameter (β) indicates the reliability through the slope of graphs. A narrower distribution results in a steeper slope. F-GNP composites show higher shape parameter values both in air aged and oil aged. Due to improved dispersibility and compatibility, F-GNP composites demonstrate higher reliability compared to P-GNP composites.

References

[1] Bidabadi, B.S; de Castro, E.M; Carrola, M.; Koirala, P.; Tehrani, M.; Asadi, A. Engineering the crystalline architecture for enhanced properties in fast-rate processing of Poly(ether ether ketone) (PEEK) nanocomposites. *ACS Appl. Eng. Mater.* **2024**, *2*, 2038-2054.

[2] Xu, D.; Gao, Y.; Sun, Y.; Wang, Z.; Jiang, Z.; Jiang, X.; Zhang, H. A novel graphene nanoplatelets (GNPs) dispersant: polyaryletherketones with pendent pyrene groups, *Macromol. Chem. Phys.* **2019**, *220*, 1800553. https://doi.org/10.1002/macp.201800553.

[3] Hung, C.Y.; Wang, C.C; Chen, C.Y. Enhanced the thermal stability and crystallinity of polylactic acid (PLA) by incorporated reactive PS-b-PMMA-b-PGMA and PS-b-PGMA block copolymers as chain extenders, *Polymer.* **2013**, *54*, 1860-1688. http://dx.doi.org/10.1016/j.polymer.2013.01.045.

[4] Mindivan, F. Effect of graphene nanoplatelets (GNPs) on tribological and mechanical behaviors of polyamide 6 (PA6), *Tribol. Ind.* **2017**, *39*, 277-282. http://doi.org/10.24874/ti.2017.39.03.01.

[5] Carotenuto, G.; Nicola, S.D.; Palomba, M.; Pullini, D.; Horsewell, A.; T.W. Hansen, Nicolais, L. Mechanical properties of low-density polyethylene filled by graphite nanoplatelets, *Nanotechnol.* **2012**, *23*, 485705. http://doi.org/10.1088/0957-4484/23/48/485705.
